# Heptageniidae (Insecta, Ephemeroptera) of Thailand

**DOI:** 10.3897/zookeys.272.3638

**Published:** 2013-02-26

**Authors:** Boonsatien Boonsoong, Dietrich Braasch

**Affiliations:** 1 Animal Systematics and Ecology Speciality Research Unit (ASESRU), Department of Zoology, Faculty of Science, Kasetsart University, Bangkok, Thailand 10900; 2 D-14471 Potsdam, Kantstraße 5, Germany

**Keywords:** Mayflies, Heptageniidae, Thailand, key

## Abstract

Nine genera and twenty-two species of heptageniid mayflies from Thailand are defined in this present work as well as one suggested further subgenus, *Compsoneuria (Siamoneuria) kovaci* (species “incertae sedis”) including some particular characters. Taxonomic remarks, diagnoses, line drawings of key characters, distribution, habitat and biological data, and a larval key to the genera and species are provided. The chorionic eggs of eight genera and eight species were observed and shown using a scanning electron microscope.

## Introduction

Heptageniidae is a family of mayflies with around 509 described species and distributed mainly in the Holoarctic, Oriental, and Afrotropical regions (Barber-James et al. 2008). Wang and McCafferty (2004: Part I) “analyzed the generic relationships and presented a phylogenetic classification of the family” while Webb and McCafferty (2008: Part II) defined the genera providing an illustrated key. Following Webb and McCafferty (2008), only 16 genera but more than 150 species of Heptageniidae can be found in the Oriental region (Soldán, 2001).

Heptageniid mayflies are one of the most abundant and common components of benthic communities in Thai running waters. The larvae inhabit slow to fast flowing streams where they occur on the surface of rocks, logs, vegetation, and leaves. Many heptageniid species have been used as indicators of anthropogenic disturbance because they are relatively intolerant of pollution change and as sensitive indicators of organic pollution (Hilsenhoff 1988) and metal pollution (Courtney and Clements 1998, Deacon et al. 2001, Clements 2004, Clark and Clements 2006). Furthermore, individuals of this family were test subjects of toxicity and drift behavior studies (Diamond et al. 1992, Céréghino et al. 2004, Stitt et al. 2006).

Heptageniidae have been recorded from Thailand by Polhemus and Polhemus (1988), Braasch (1990), Sites et al. (2001), Sangpradub et al. (2002), Wang and McCafferty (2004), Braasch (2006a), Webb and McCafferty (2006), and recently by Braasch and Boonsoong (2009, 2010). However, taxonomic revision of the family Heptageniidae in Thailand is urgently needed, because the study of life stages is still in its infancy. This is mainly due to problems of identification, unsettled generic questions, and the lack of use of modern genetic methods to construct a phylogeny of the family Heptageniidae from Southeast Asia. In this paper, we provide a larval key to known genera and species of Thai heptageniid mayflies, with particular emphasis on the problems of identification of several species. Taxonomic remarks, diagnoses, line drawings of key characters, distribution, and habitat and biological data are provided. In addition, the egg chorions of eight Thai heptageniid species were observed. All of the egg specimens used in this study were obtained from mature larvae and adults. The material was first preserved in alcohol and then critical-point dried using carbon dioxide and finally placed on holders and coated with gold. The oological observations of eight heptageniid species were made with a JEOL JSM-5600LV scanning electron microscope. The terminology provided by Koss and Edmunds (1974) is used in this paper.

In the following key and text, abbreviations are as follows: alt (altitude), asl (above sea level), μS/cm (microsiemens per centimeter), comb. (combination), M (male imago), F (female imago), mount. (mountainous), Ms (male subimago), Fs (female subimago), NP (National Park), orig. (original), sec. (second), WS (Wildlife Sanctuary).

## A Larval Key to the Genera, Subgenera and Known Species of Heptageniidae in Thailand

**Table:** 

1	Median caudal filament absent (Fig. 1A, 9D)	*Epeorus*, 2
–	Median caudal filament present	8
2	Lamellae of gills 2-7 with anal ribs arched (Fig. 1B)	subgenus *Belovius*, 4
−	Lamellae of gills 2-7 without anal ribs arched	3
3	Lamellae of gills 1 greatly extended beneath the abdomen (Fig. 1C–D)	subgenus *Iron*, 5
–	Lamellae of gills 1 somewhat extended beneath the abdomen (Fig. 1I)	subgenus *Epeorus*, 6
4	Abdominal terga 2–9 each with long, acute median spine on posterior margin (Fig. 1F)	*Epeorus unicornutus* Braasch
–	Abdominal terga 2–9 each without acute median spine on posterior margin (Fig. 1G)	*Epeorus khayengensis* Boonsoong & Braasch
5	Abdominal terga with paired long acute submedian spines	*Epeorus martinus* Braasch & Soldán
–	Abdominal terga without paired acute submedian spines; foretibiae relatively long; median dark brown on the abdominal terga	*Epeorus thailandensis* Braasch & Boonsoong
6	Pair of submedian spines on terga 2-9 relatively long (Fig. 1A)	*Epeorus aculeatus* Braasch
–	Pair of submedian spines on terga 2-9 relatively short	7
7	Paired tubercles on terga rounded, blunt bristles densely rowed	*Epeorus bifurcatus* Braasch & Soldán
−	Paired tubercles on terga more rounded, pointed bristles (Fig. 1H, 1J)	*Epeorus inthanonensis* Braasch & Boonsoong
8	Gill-pairs 1 meet or overlap ventrally to form a friction disc (Fig. 2A)	*Rhithrogena siamensis* Braasch & Boonsoong
–	Gill-pairs 1 not meeting ventrally and not forming a ventral friction disc	9
9	Lamellae of gills 1 minute, lamellae of gills 2-7 long, narrow and sharply pointed (Fig. 2B–E); ventral surface of maxillae with setae in row (Fig. 2F); abdominal terga with fan-shaped robust setae (Fig. 2G)	*Trichogenia maxillaris* Braasch & Soldán
–	Lamellae of gills 1 similar in shape and size to other gills; gill lamellae usually not as above; ventral surface of maxillae with scattered setae (Fig. 2H); abdominal terga with fine setae only	10
10	Abdominal terga with median dorsal ridge (Fig. 2I–J); claws with denticles	*Notacanthurus baei* Braasch & Boonsoong
–	Abdominal terga without median dorsal ridge	11
11	Supracoxal sclerites rounded or bluntly pointed (Fig. 3A)	15
–	Supracoxal sclerites sharply pointed (Fig. 3B)	12
12	Anterior margin of head capsule distinctly thickened (Fig. 3C); posterolateral spines of abdomen well developed (Fig. 3D)	*Thalerosphyrus sinuosus* Navás
–	Anterior margin of head capsule not thickened; dorsal view of abdomen; posterolateral spines of abdomen small (Fig. 3I, K)	*Compsoneuria*, 13
13	Shape of gills 3-6 without emarginations	14
–	Shape of gills 3-6 with emarginations (Fig. 3E–H)	*Compsoneuria (Siamoneuria) kovaci* Braasch
14	Dorsal view of abdomen as Fig. 3I; shape of gill 7 leaf-like and pointed apically (Fig. 3J)	*Compsoneuria thienemanni* Ulmer
–	Dorsal view of abdomen as Fig. 3K; shape of gill 7 lanceolate and rounded apically (Fig. 3L)	*Compsoneuria langensis* Braasch & Boonsoong
15	Gills 7 slender and pointed (Fig. 4A); robust setae on inner surface of hindtarsi pectinate (Fig. 4B)	*Asionurus primus* Braasch & Soldán
–	Gills 7 usually rounded apically, never as long and narrow as above; setae on inner surface of tarsi either simple or fimbriate, never pectinate	16
16	Cerci bear spines as well as lateral bristles and segments of the cerci with stout spines alternate with those lacking such spines (Fig. 4C); gills 1–7 with row of sparse marginal set	*Rhithrogeniella tonkinensis* Soldán & Braasch
–	Cerci not as above (Fig. 4D); gills 1–7 without row of sparse marginal setae	*Afronurus*, 17
17	Body and head with indistinct large pale dots and markings dorsally	18
–	Body and head with distinct large pale dots and markings dorsally (Fig. 4E)	19
18	Gills 1 banana-shaped	*Afronurus namnaoensis* Braasch & Boonsoong
–	Gills 1 symmetrically pointed ovaloid (Fig. 4F)	*Afronurus gilliesiana* Braasch
19	Gills 7 unsymmetrically ovaloid, obtusely pointed apically	*Afronurus rubromaculatus* You, Wu, Gui & Hsu
–	Gills 7 narrowly lanceolate (Fig. 4G)	*Afronurus rainulfiana* Braasch

## Taxonomic descriptions

### Genus Epeorus

Eaton, 1881

http://species-id.net/wiki/Epeorus

Figs 1A–J 5A–B 9D

#### Remarks.

*Epeorus* is widely distributed in the Holarctic, northern portion of the Neotropical, Palaearctic, and Oriental regions (Kluge 2004). In tropical Southeast Asia, species of this genus have been reported and described by Braasch (1990, 2011), Braasch and Boonsoong (2010), Braasch and Soldán (1979, 1984c), and Webb and McCafferty (2006). Nguyen and Bae (2004a) provided larval descriptions and a key to six species of Vietnamese *Epeorus* ; this was the first comprehensive taxonomic study of the larvae of *Epeorus* from tropical Southeast Asia. The first record of *Epeorus* in Thailand is of *Epeorus aculeatus* Braasch (1990) from Doi Inthanon National Park, Chiang Mai province. Recently, the first Thai imago of *Epeorus aculeatus* was described by Webb and McCafferty (2006) from Chiang Mai province. Originally, *Epeorus unicornutus* was recorded from Himalayas (Braasch 2006b) although it had been already collected in Thailand December 1987/1998 from the river Nam Lang, Soppong / Pangmapa, Mae Hong Son province (Braasch, unpublished). Currently, seven species of *Epeorus* are known from Thailand (Braasch and Boonsoong 2010).

**Figure 1.**
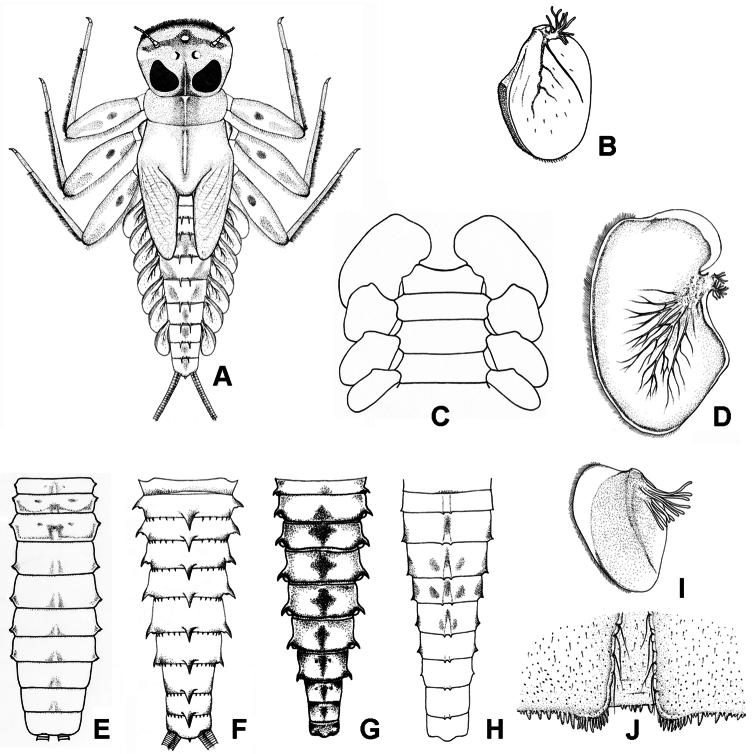
**A** Habitus of *Epeorus aculeatus* Braasch, 1990 **B** lamellae of gills 7 of *Epeorus khayengensis* Boonsoong & Braasch, 2010 **C–E** ventral view of abdomen (**C**), abdominal gills 1 (**D**) and abdominal terga of (**E**) *Epeorus thailandensis* sp. n. **F** abdominal terga of *Epeorus unicornutus* Braasch, 2006 **G** abdominal terga of *Epeorus khayengensis* Boonsoong & Braasch, 2010 **H–J** abdominal terga (**H**), lamellae of gills 1 (**I**) and tergum VII (**J**) of *Epeorus inthanonensis* Braasch & Boonsoong, 2010.

### Epeorus (Belovius) khayengensis

Boonsoong & Braasch, 2010

http://species-id.net/wiki/Epeorus_khayengensis

Figs 1B, 1G 5A–B 9D

Epeorus (Belovius) khayengensis Boonsoong & Braasch, 2010: 13–17, Figs 53–68. (orig.)

#### Larva.

Braasch and Boonsoong 2010: 13-17, Figs 53–68.

#### Adult.

Unknown.

#### Eggs.

Egg chorion of *Epeorus khayengensis* very smooth without any peculiar structure (Fig. 5A), 2-3 micropyles visible in the equatorial area (Fig. 5B).

#### Distribution.

Huai Khayeng stream (Thong Pha Phum district, Kanchanaburi province).

#### Diagnosis.

The larva of *Epeorus khayengensis* (Fig. 9D) can be distinguished from that of othercongeners by abdominal terga 2–9 without long acute median spine on posterior margin, but bearing long hair-like setae.

#### Habitat and biology.

The larva of *Epeorus khayengensis* inhabits tropical headwater streams approximately 210 m in alt. The streams range between 6–7 m in width and 10–11 cm in depth. The water temperature ranges between 22–25°C, pH between 6.35–7.15, total dissolved solids are between 27–34 mg/L, and conductivity is between 41–53 µS/cm. The larvae are found in eroded areas of streams where moderately flowing over cobble and sandy bottom.

#### Remarks.

Braasch and Boonsoong (2010) described this species from Thailand based on nymphal specimens, and deposited them in the Zoological Museum, Kasetsart University (ZMKU), Bangkok. The adults of *Epeorus khayengensis* are unknown.

### Epeorus (Belovius) unicornutus

Braasch, 2006

http://species-id.net/wiki/Epeorus_unicornutus

Fig. 5F

Epeorus (Belovius) unicornutus Braasch, 2006: 80, 82, Figs 1–8. (orig.)

#### Larva.

Braasch 2006b: 80, 82, Figs 1–8.

#### Adult.

Unknown.

#### Eggs.

Unknown.

#### Distribution.

Nam Thob Ranger Station, Phu Luang Wildlife Sanctuary, Nam Thob stream (Loei province).

#### Diagnosis.

The larva of *Epeorus unicornutus* can be distinguished from that of other congeners by the combination of the following characters: abdominal terga 2-9 each with single, prominent, acute median spine and with a row of short spines on posterior margin, tergum 10 with short spines and hair-like setae on posterior margin.

#### Habitat and biology.

Larvae of *Epeorus unicornutus* are found in headwater streams shaded under tree canopies in mountainous areas (alt 330 m) where the streams are 10–12 m wide and 10–15 cm in depth. The water temperature ranges between 22–23°C, pH between 7.0–7.2, total dissolved solids range between 18–20 mg/L, and conductivity between 28–30 µS/cm. The larvae are found underneath stones in fast flowing reaches of the streams. The coarse mineral substrate consists of boulder (60%), cobble (30%), gravel and coarse sand (10%), and abundant fallen leaves. *Epeorus unicornutus* is found under minimally disturbed conditions of Nam Thob streams, Loei province.

#### Remarks.

Braasch (2006b) described this species based on larval specimens from River Indravati near Dalaghat 1,200 m, Nepal, Himalayas. In this study, this species were found from northern and northeastern parts of Thailand. This species is different from other known species of the genus *Epeorus* by abdominal terga 2-9 each with single, prominent, and acute median spine.

### Epeorus (Iron) martinus

Braasch & Soldán, 1984

http://species-id.net/wiki/Epeorus_martinus

Iron martinus Braasch & Soldán, 1984: 113-114, Figs 35–44. (orig.)
Epeorus (Iron) martinus Braasch & Soldán, 1984 (comb.)

#### Larva.

Braasch and Soldán 1984c: 113–114, Figs 35–44; Nguyen and Bae 2004a: 102–104, Figs 1–6.

#### Adult.

Unknown.

#### Eggs.

Unknown.

#### Distribution.

Khun Kon Waterfall (Chiang Rai province).

#### Diagnosis.

The larva of *Epeorus martinus* can be distinguished by the following characteristics: pairs of moderately long acute submedian spines on the abdominal terga 1-9, large but narrow gill 1 forming a sucking disc and gill 7 being unfolded.

#### Habitat and biology.

*Epeorus martinus* larvae are found in mountain streams with a moderate current, at an elevation of 300–2800 m (Nguyen and Bae 2004a).

#### Remarks.

Webb and McCafferty (2008) did not recognize the subgenera of *Epeorus* (e.g. *Belovius*, *Iron*). However, we hold on the validity of subgenus *Belovius* and *Iron* within genus *Epeorus* (Boonsoong & Braasch, 2010). Therefore, we propose *Iron martinus* Braasch & Soldán, 1984 = *Epeorus (Iron) martinus* Braasch & Soldán, 1984

### Epeorus (Iron) thailandensis

Braasch & Boonsoong sp. n.

urn:lsid:zoobank.org:act:1BCDC6C6-F254-4C28-ABBB-CF520AA3A2E4

http://species-id.net/wiki/Epeorus_thailandensis

Figs 1C–E

Iron longitibius Nguyen & Bae, 2004 (questionable): 20–22, Figs 83–98.
Epeorus (Iron) thailandensis Braasch & Booonsoong sp. n.

#### Larva.

Braasch and Boonsoong (2010) have described new species sub nom. “*Epeorus (Iron) longitibius* Nguyen & Bae, 2004 (questionable)”: 20–22 (Figs 83–98). The deposition of the female larva is in ZMKU (80% alcohol).

#### Adult.

Unknown.

#### Eggs.

Unknown.

#### Distribution.

Region of Doi Inthanon (Chiang Mai province); alt 2000 m, III 1999; bottom sample (leg. R. Braasch).

#### Diagnosis.

In contrast tothe larva of *Epeorus longitibius* with foretibiae 1.2 × length of forefemora, *Epeorus thailandensis* has foretibia of equal length; forefemur of new species is with a small femoral spot, *Epeorus longitibius* without such one; the gills 2-6 are quadrangular and gill 7 has a fold in *Epeorus thailandensis*, but in *Epeorus longitibius* gills 2-6 are elongated and gill 7 unfolded.

#### Habitat and biology.

The larvae of *Epeorus thailandensis* live presumably in high mountain streams with high oxygen concentrations and a faster current where the substrate is mostly stony at an elevation of 2000 m.

#### Remarks.

Boonsoong and Braasch (2010) misidentified this species as *Iron longitibius* Nguyen & Bae, 2004 (questionable). In this study, we have re-checked them and found , then we re-identified it as a new species, *Epeorus (Iron) thailandensis*. The new species is different from the larva of *Iron longitibius* by characters within length of foretibia, femoral spot, and gill shape of gills 2–7. In this observation, we propose as the new species.

### Epeorus (Epeorus) aculeatus

Braasch, 1990

http://species-id.net/wiki/Epeorus_aculeatus

Fig. 1A

Epeorus aculeatus Braasch, 1990: 7-9, Figs 1–8. (orig.)
Epeorus (Epeorus) aculeatus Braasch, 1990 (comb.)

#### Larva.

Braasch 1990: 7–9, Figs 1–8; Nguyen and Bae 2004a: 19–21, Figs 1–6.

#### Adult.

**Figure 2.**
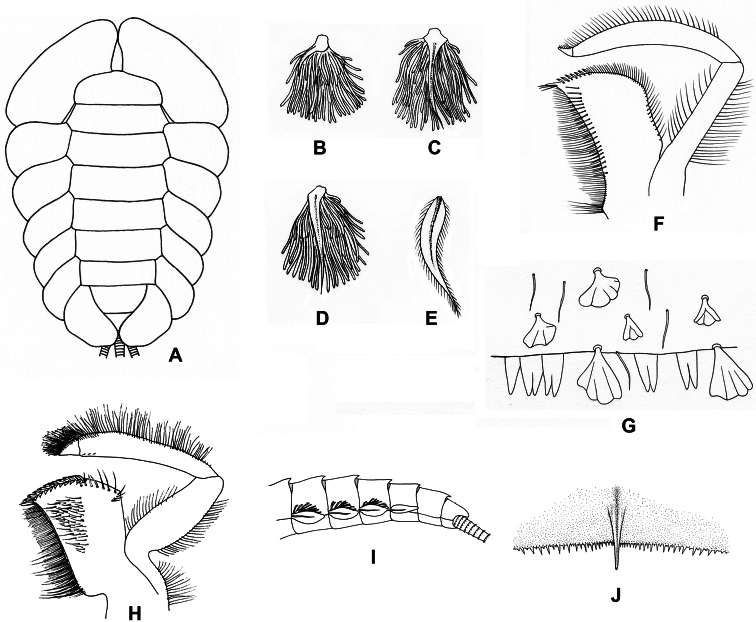
**A** Ventral view of abdomen of *Rhithrogena siamensis* Braasch & Boonsoong, 2009 **B–E** lamellae of gills 1 (**B**), 3 (**C**), 5 (**D**) and 7 (**E**) of *Trichogenia maxillaris* Braasch & Soldán, 1988 **F** ventral view of left maxilla of *Trichogenia maxillaris* Braasch & Soldán, 1988 **G** bristles on dorsal face of abdominal terga of *Trichogenia maxillaris* Braasch & Soldán, 1988 **H** ventral view of left maxilla of *Compsoneuria langensis* Braasch & Boonsoong, 2010 **I-J** abdominal terga (**I**) and tergum VII (**J**) of *Notacanthurus baei* Braasch & Boonsoong, 2009.

**Figure 3.**
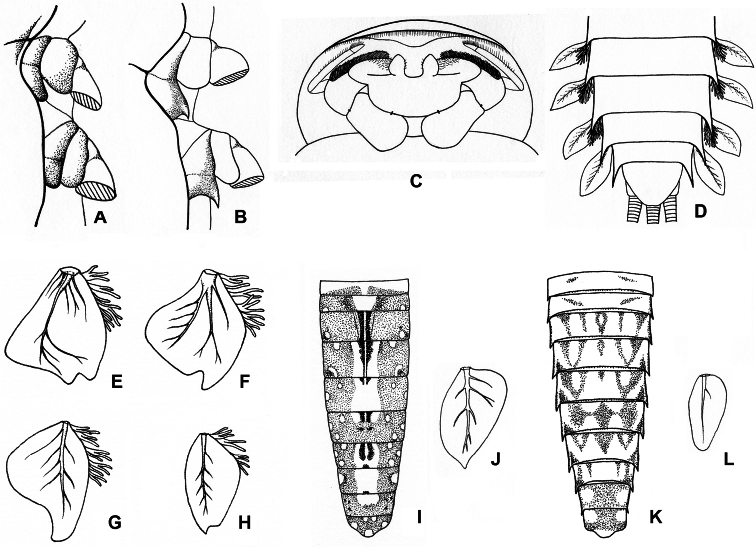
**A** Right side of thorax of *Afronurus namnaoensis* Braasch & Boonsoong, 2010 **B–D** right side of thorax (**B**), ventral view of head capsule (**C**) and ventral view of posterior abdomen (**D**) of *Thalerosphyrus sinuosus* Navás, 1933 **E–H** lamella of gills 3 (**E**), 4 (**F**), 5 (**G**), 6 (**H**) of *Compsoneuria (Siamoneuria) kovaci* Braasch, 2006 **I–J** dorsal view of abdomen (**I**), lamella of gills 7 (**J**) of *Compsoneuria thienemanni* Ulmer, 1939 **K–L** dorsal view of abdomen (**K**), lamella of gills 7 (**L**) of *Compsoneuria langensis* Braasch & Boonsoong, 2010.

**Figure 4.**
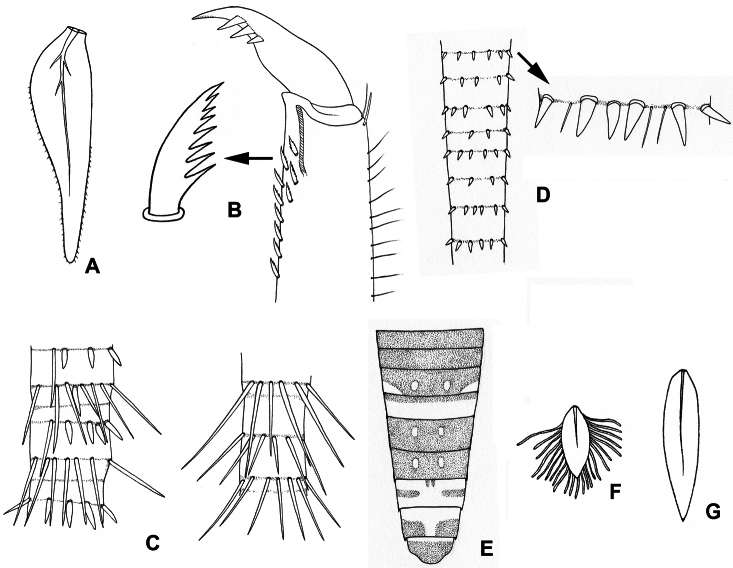
**A–B** Lamella of gills 7 (**A**) and setae on inner surface of hind tarsi (**B**) of *Asionurus primus* Braasch & Soldán, 1986 **C** bristles on cerci of *Rhithrogeniella tonkinensis*Soldán & Braasch, 1986 **D** bristles on cerci of *Asionurus namnaoensis* Braasch & Boonsoong, 2010 **E** dorsal view of abdomen of *Asionurus rubromaculatus* You, Wu, Gui & Hsu, 1981 **F** lamella of gills 1 of *Asionurus gilliesiana* Braasch, 1990 **G** lamella of gills 7 of *Asionurus rainulfiana* Braasch, 1990**. **

**Figure 5.**
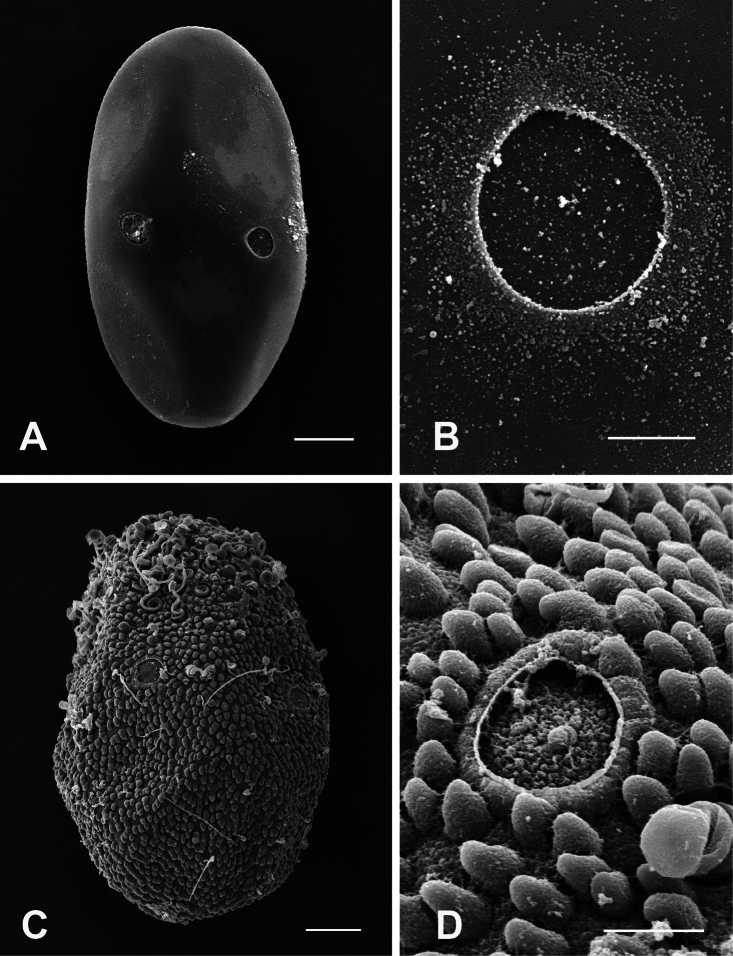
**A–B** General outline (**A**) and micropyle (**B**) of the egg of *Epeorus khayengensis* Boonsoong & Braasch, 2010 **C-D** General outline (**C**) and micropyle (**D**) of the egg of *Rhithrogena siamensis* Braasch & Boonsoong, 2009. Scale bars 20 µm for **A** and **C**; 5 µm for **B** and **D**.

Webb and McCafferty 2006: 65–68, M, Figs 1–5.

#### Eggs.

Unknown.

#### Distribution.

Mae Chaem district, Doi Inthanon NP (Chiang Mai province); Braasch (1990) recorded *Epeorus aculeatus* from Doi Inthanon National Park, Chiang Mai province.

#### Diagnosis.

The larva of *Epeorus aculeatus*can be distinguished from that of othercongeners by abdominal terga 2-9 (Fig. 1A) each bearing a pair of long, acute, submedian spines on its posterior margin, and with a median dark brown spot on femoral surface.

#### Habitat and biology.

The larva of *Epeorus aculeatus* occurs in headwater streams between 600–740 m alt in Thailand and high mountain streams between 1,400-2,800 m in Vietnam (Nguyen and Bae 2004a). The larvae are mostly found under rocks in fast flowing reaches of the streams. The substrate consists of mixed sand/gravel and larger stones such as boulders and cobble.

#### Remarks.

Braasch (1990) described this species based on larval specimens. Then, Webb and McCafferty (2006) described the male imago of *Epeorus aculeatus* based on reared Thai specimens from Doi Suthep NP, Chiangmai province. This species is different from other known species of the genus *Epeorus* by each bearing a pair of long, acute, submedian spines on its posterior margin.

### Epeorus (Epeorus) bifurcatus

Braasch & Soldán, 1979

http://species-id.net/wiki/Epeorus_bifurcatus

Epeorus bifurcatus Braasch & Soldán, 1979: 266, 270, Figs 15–22. (orig.)
Epeorus (Epeorus) bifurcatus Braasch & Soldán, 1979 (comb.)

#### Larva.

Braasch and Soldán 1979: 266, 270, Figs 15–22; Nguyen and Bae 2004a: 21–22, Figs 7–12.

#### Adult.

Unknown.

#### Eggs.

Unknown.

#### Distribution.

Tak province, highway 1090, km 64.5, mountain creek, riffle and run habitats; limy, gravel; leaf packs, wood, secondary forest; 750 m asl, c 16°30'N, 99°00'E; 14.1.2009 (leg. Freitag).

#### Diagnosis.

The larva of *Epeorus bifurcatus* can be distinguished from that of itscongeners by pairs of small submedian dorsal tubercles on tergites with acute spines and gill 1 larger than gill 3. In the Figs 7–8 of Nguyen and Bae (2004a) some characters (the femoral spot, the rim of hairs of the hind margin of head and pronotum) are not described. Furthermore, according to our material, the head of the type species is smoothly trapezoid without markings in anterior median half, however, the head is transversely ellipsoid with a broad dark band between its front and hind margins.

#### Habitat and biology.

Nguyen and Bae (2004a) noted that the larva of *Epeorus bifurcatus* occur in mountain streams ranging between 200–600 m. They were found on the underside of stones in fast flowing sections of the streams.

#### Remarks.

Braasch and Soldán (1979) described this species based on larval specimens and original descriptions are written in Germany. Then, Nguyen and Bae (2004a) described the larva of *Epeorus bifurcatus* in English based on specimens from northern Vietnam, near the holotype locality.

### Epeorus (Epeorus) inthanonensis

Braasch & Boonsoong, 2010

http://species-id.net/wiki/Epeorus_inthanonensis

Figs 1H–J

Epeorus inthanonensis Braasch & Boonsoong, 2010: 17–19, Figs 69–82. (orig.)
Epeorus (Epeorus) inthanonensis Braasch & Boonsoong, 2010 (comb.)

#### Larva.

Braasch and Boonsoong 2010: 17–19, Figs 69–82.

#### Adult.

Unknown.

#### Eggs.

Unknown.

#### Distribution.

Doi Inthanon NP (Chiang Mai province).

#### Diagnosis.

This species resembles *Epeorus bifurcatus* however, the larva of *Epeorus inthanonensis* has paired tubercles on the terga which are more rounded than in *Epeorus bifurcatus*; they lack spines and gill 1 is smaller than gills 2–6.

#### Habitat and biology.

Braasch and Boonsoong (2010) noted that the larva of *Epeorus inthanonensis* wasfound in small mountain streams ranging between 800–2000 m.

#### Remarks.

This species was described by Braasch and Boonsoong (2010). This species resembles *Epeorus bifurcatus* however, the larva has paired tubercles on the terga which are more rounded than in *Epeorus bifurcatus***.**

### Genus Rhithrogena

Eaton, 1881

http://species-id.net/wiki/Rhithrogena

Figs 2A 5B–D

#### Remarks.

The genus *Rhithrogena* is the most diverse in the Holarctic, with numerous species also in the Palaearctic Asia. However, it seems to be under-representedinSoutheast Asia. Braasch and Boonsoong (2009) described *Rhithrogena siamensis* Braasch & Boonsoong, 2009 from northern Thailand, this species also occurs in the northeastern and western parts of Thailand. Only two further species of the genus can be found in this area: *Rhithrogena parva* Ulmer, 1939 from Taiwan (Ulmer 1912) and *Rhithrogena diehliana* Braasch & Soldán, 1986 from Sumatra (Braasch and Soldán 1986c).

### Rhithrogena (Tumungula) siamensis

Braasch & Boonsoong, 2009

http://species-id.net/wiki/Rhithrogena_siamensis

Figs 2A 5C–D

Rhithrogena (Tumungula) siamensis Braasch & Boonsoong, 2009: 39–43, Figs 32–47. (orig.)

#### Larva. 

Braasch and Boonsoong 2009: 39–43, Figs 32–47.

#### Adult.

Braasch and Boonsoong 2009: 38-39, M, Figs 19–30; F, Fig. 31.

**Eggs.** General shape ovoid. One of the poles terminates with large knob-terminated coiled threads (KCTs) (Fig. 5C), the whole chorion is covered with uniform granules and scattered with small loose KCTs, with a large micropyle on the equatorial plane (Fig. 5D).

#### Distribution.

Mae Hong Son province, Chiang Mai province, Chiang Rai province, Loei province.

#### Diagnosis.

The larva of *Rhithrogena siamensis* resembles *Rhithrogena (Tumungula) unica* Zhou & Peters, 2004 but differs in mouthparts structure and gill 1, with *Rhithrogena siamensis* being pointed-crenulate and having longer plica which are more bluntly rounded, whereas that of *Rhithrogena (Tumungula) unica* has a few angular crenulations and a shorter, more strongly rounded plica.

#### Habitat and biology.

*Rhithrogena siamensis* larvae cling to rock surfaces in medium- to fast-flowing water. Collections over most of the year revealed that the flight season at altitudes of 600 m was mainly during March/April, just before the beginning of the monsoon rains in May.

#### Remarks.

Only one species of *Rhithrogena* was identified in our study as being distributed thoughout Thailand. They live in a rapid current of stream.

### Genus Trichogenia

Braasch & Soldán, 1988

http://species-id.net/wiki/Trichogenia

Figs 2B–G

#### Remarks.

The Southeast Asian genus *Trichogenia* was established by Braasch and Soldán (1988) from Vietnam. Four species of *Trichogenia* are present in the Oriental region (Webb et al. 2006). Onlyone species, so far, is found in northern and northeastern Thai streams.

### Trichogenia maxillaris

Braasch & Soldán, 1988

http://species-id.net/wiki/Trichogenia_maxillaris

Figs 2B–G

Trichogenia maxillaris Braasch & Soldán, 1988: 119-124, Figs 1–13. (orig.)
Heptagenia maxillaris Kluge, 2004: 173. (comb.)

#### Larva.

Braasch and Soldán 1988: 119-124, Figs 1–13.

#### Adult.

Unknown.

#### Eggs.

Unknown.

#### Distribution.

Doi Suthep, Chiang Mai province; Loei province; Soppong, Mae Hong Son province.

#### Diagnosis.

*Trichogenia maxillaris* can be differentiated from congeners by the following combination of characteristics: gill lamellae 2–7, long, narrow and sharply pointed; base of outer canines of mandibles without dense lateral brush of setae; supracoxal sclerites short. The other *Trichogenia* species have gill lamellae 5-7 rounded with a pointed apex, base of outer canines of mandibles with dense lateral brush of setae, and supracoxal sclerites long and pointed.

#### Habitat and biology.

*Trichogenia maxillaris* larvae occur in small mountain streams. To date, only a few *Trichogenia* specimens have been collected in Thai streams. This species appears to be a sensitive indicator because larvae were found exclusively in forest stream areas.

#### Remarks.

Only one species of *Trichogenia* (*Trichogenia maxillaries*) was reported from Thailand. The larva of this species was described by Braasch and Soldán (1988). The adults are unknown.

### Genus Notacanthurus

Tshernova, 1974

http://species-id.net/wiki/Notacanthurus

Figs 2I–J 6A–B

#### Remarks.

Three species of *Notacanthurus* are described from the Himalayas (Braasch 1980, 1986). The Thai species *Notacanthurus baei* Braasch & Boonsoong, 2009was collected and described from the northern part of country.

### Notacanthurus baei

Braasch & Boonsoong, 2009

http://species-id.net/wiki/Notacanthurus_baei

Figs 2I–J 6A–B

Notacanthurus baei Braasch & Boonsoong, 2009: 34–38, Figs 1–18. (orig.)

#### Larva.

Braasch and Boonsoong 2009: 34–38, Figs 1–18.

#### Adult.

**Figure 6.**
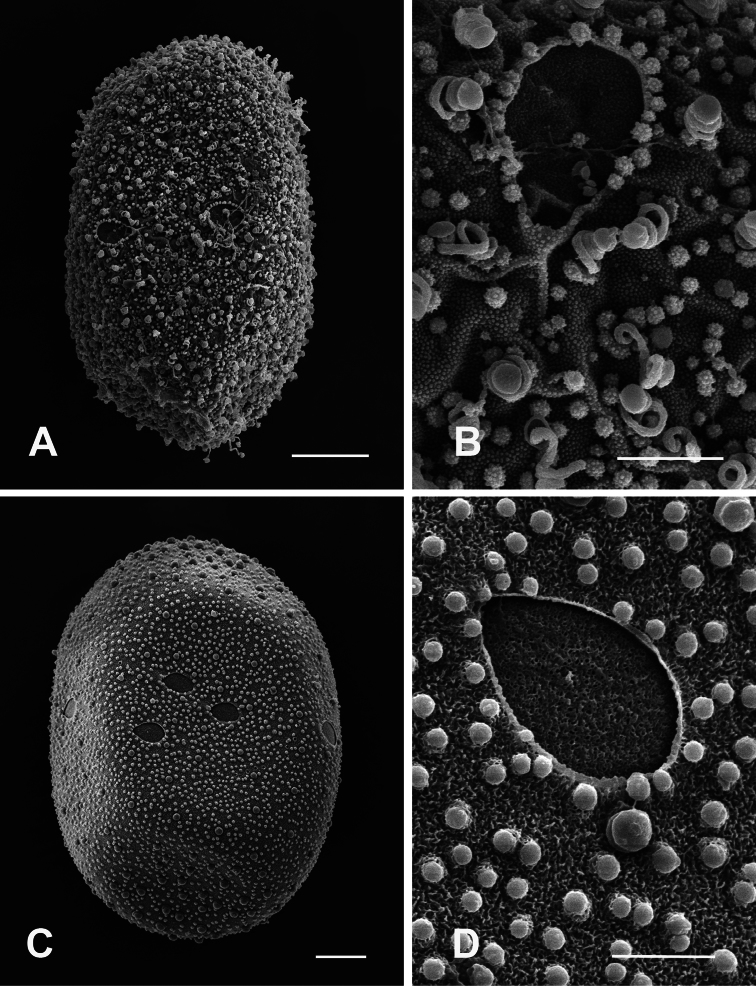
**A–B** General outline (**A**) and micropyle (**B**) of the egg of *Notacanthurus baei* Braasch & Boonsoong, 2009 **C-D** General outline (**C**) and micropyle (**D**) of the egg of *Thalerosphyrus sinuosus* Navás, 1933. Scale bars 20 µm for **A** and **C**; 5 µm for **B** and **D**.

**Figure 7.**
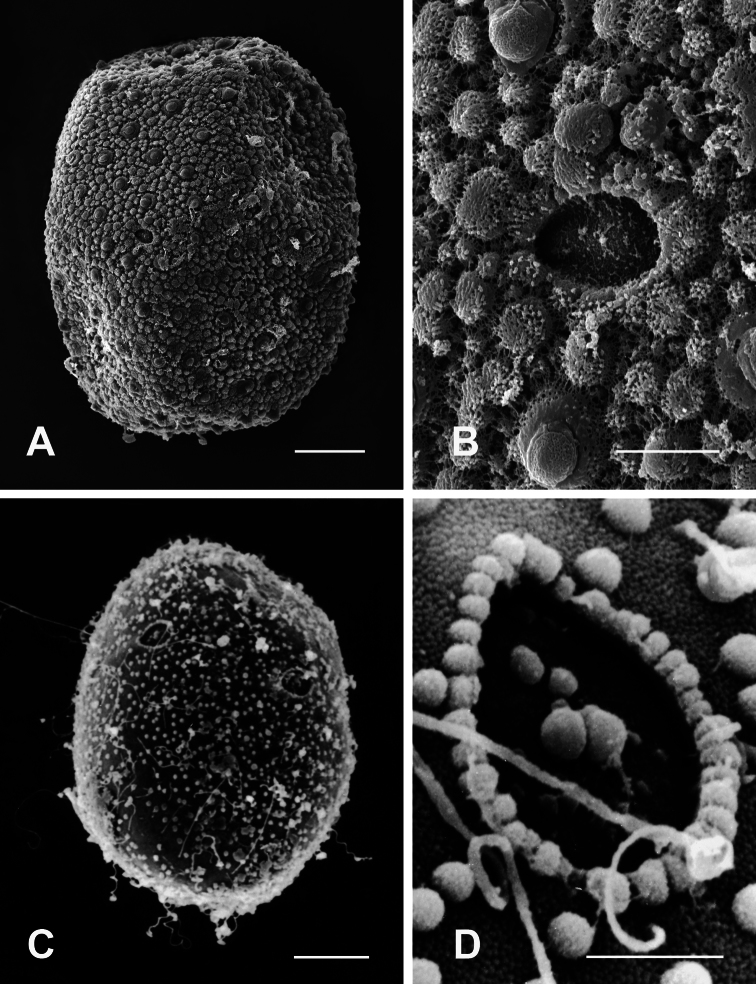
**A–B** General outline (**A**) and micropyle (**B**) of the egg of *Compsoneuria thienemanni*Ulmer, 1939 **C–D **General outline (**C**) and micropyle (**D**) of the egg of *Asionurus primus* Braasch & Soldán, 1986. Scale bars 20 µm for **A** and **C**; 5 µm for **B** and **D**.**<br/>**

**Figure 8.**
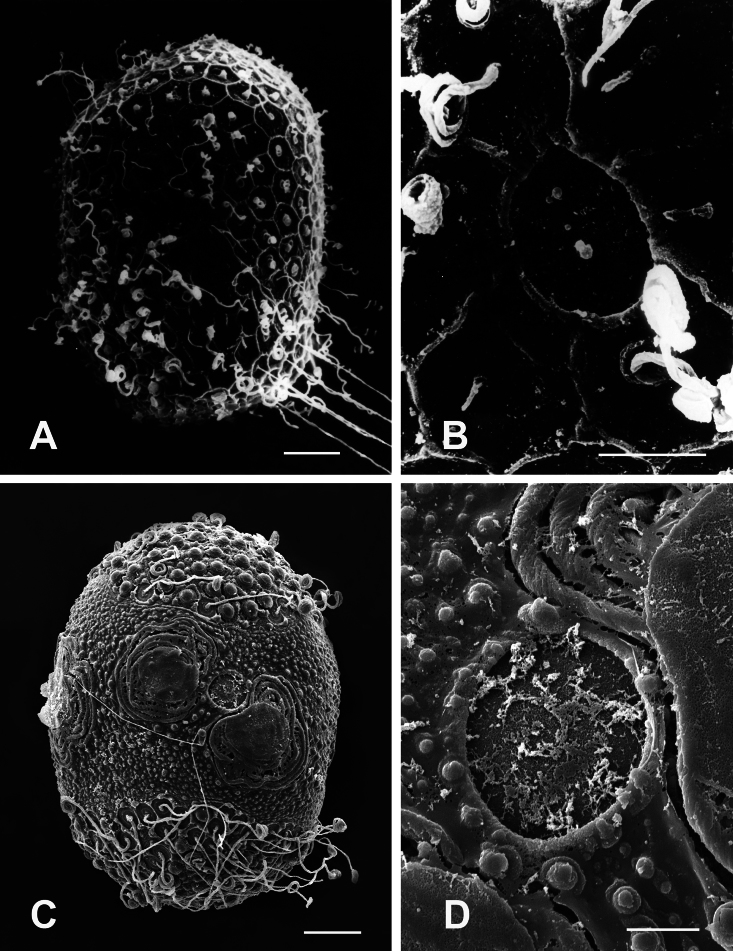
**A–B** General outline (**A**) and micropyle (**B**) of the egg of *Rhithrogena tonkinensis*Soldán & Braasch, 1986 **C–D** General outline (**C**) and micropyle (**D**) of the egg of *Asionurus namnaoensis* Braasch & Boonsoong, 2010. Scale bars 20 µm for **A** and **C**; 5 µm for **B** and **D**.

Unknown. Its prospective penis (Fig. 14, Braasch 1986) is quite unlike the bilobed penes of the Himalayan *Notocanthurus* (Figs 4-8, Braasch 1986).

#### Eggs.

Chorionic pattern of geometrically arranged small KCTs covering the entire egg surface (Fig. 6A) and interspersed among crenulated granules, folded surface of the chorion, many microgranules densely scattered all over the surface of the chorion, large micropyles on equatorial plane (Fig. 6B).

#### Distribution.

Mae Hong Son province, Mae Chaem district, Doi Inthanon NP, Doi Suthep NP (Chiang Mai province)

#### Diagnosis.

Larvae of *Notacanthurus baei* are easily identified by having a dorsal median abdominal ridge on tergites 1-9 and denticles on the claws. Larvae of the Indian species *Notocanthurus edentatus* Braasch, 1986 have no dorsal ridges on the abdomen (Braasch 1986); however, all Himalayan species of *Notacanthurus* key out by the absence of denticles on the claws. Its prospective penis (Fig. 14, Braasch 1986) is quite unlike the bilobed penes of the Himalayan *Nothacanthurus* (Figs 4–8, Braasch 1986)” and is expected to be a simple, not bilobed, penis in the male imago.

#### Habitat and biology.

*Nothacanthurus baei* larvae inhabit small streams and brooks. Larvae were usually found together with those of *Asionurus* species**.**

#### Remarks.

Braasch and Boonsoong (2009) described only one species of *Nothacanthurus* from Thailand. The adults of *Nothacanthurus baei* are unknown.

### Genus Thalerosphyrus

Eaton, 1881

http://species-id.net/wiki/Thalerosphyrus

Figs 3B–D 6C–D

#### Remarks.

*Thalerosphyrus* occurs from China through Southeast Asia to India. This genus is originally described from tropical Southeast Asia. *Thalerosphyrus sinuosus* Navás, 1933seems to be widely distributed in Thailand and adjacent countries (Nguyen and Bae 2004b). Larvae of *Thalerosphyrus* are usually the dominant heptageniids in Thai streams as well as those of *Afronurus*.

### Thalerosphyrus sinuosus

Navás, 1933

http://species-id.net/wiki/Thalerosphyrus_sinuosus

Figs 3B–D 6C–D 9F

Thalerosphyrus sinuosus Navás, 1933: 70, F, Fig. 80. (orig.)

#### Larva.

Braasch and Soldán 1984b: 203–205 (sub name *Thalerosphyrus siamensis* Dang, 1967, Figs 5–20v, 26)

#### Adult.

Navás 1933: 70, F, fig. 80; Ulmer 1939: 551–555, M&F, Figs 118–128; Braasch and Soldán 1984b: 201–206, Figs 1–8, M, 25.

#### Eggs.

KCTs randomly scattered laterally and concentrated at both poles, although larger and numerous at the pole (Fig. 6C). Rounded tubercles are scattered all over the surface of the chorion (Fig. 6D). Five – six micropyles located on equatorial plane.

#### Distribution.

Widely distributed in many parts of Thai streams.

#### Diagnosis.

The combination of having a distinctly thickened anterior margin of the head capsule, long posterolateral spines on the abdomen, acutely pointed supracoxal spurs, and well-developed lamellae on gills 1 will distinguish *Thalerosphyrus* from other Ecdyonurinae genera. Within *Thalerosphyrus* is a ‘*sinuosus*’ group of species with larvae having the above-mentioned combination, having in both sexes of adults “costal and subcostal fields with two indistict umbra-brown spots, the first at the beginning of pterostigmatic region, the second directly to the wing tip” (Navás 1933) and a ‘*determinatus*’ group, having larvae with short posterolateral spines; however, both sexes of adults have umbra-brown tinged costal and subcostal fields on forewings. In general, the species differentiation in the genus (*Thalerosphyrus* ‘*sinuosus*’ group) is unsatisfying and requires verification. The larva of *Thalerosphyrus sinuosus* can be differentiated by the combination of the following characters: gill 3 of rounded shape is much less wide than that of *Thalerosphyrus vietnamensis* (Dang, 1967) and while in *Thalerosphyrus flowersi* (Venkataraman and Sivaramakrishnan 1987) the inner side of gill 1 has a straight margin, that of *Thalerosphyrus vietnamensis* (=*Thalerosphyrus sinuosus*?) is slightly concave.

#### Habitat and biology.

**Figure 9.**
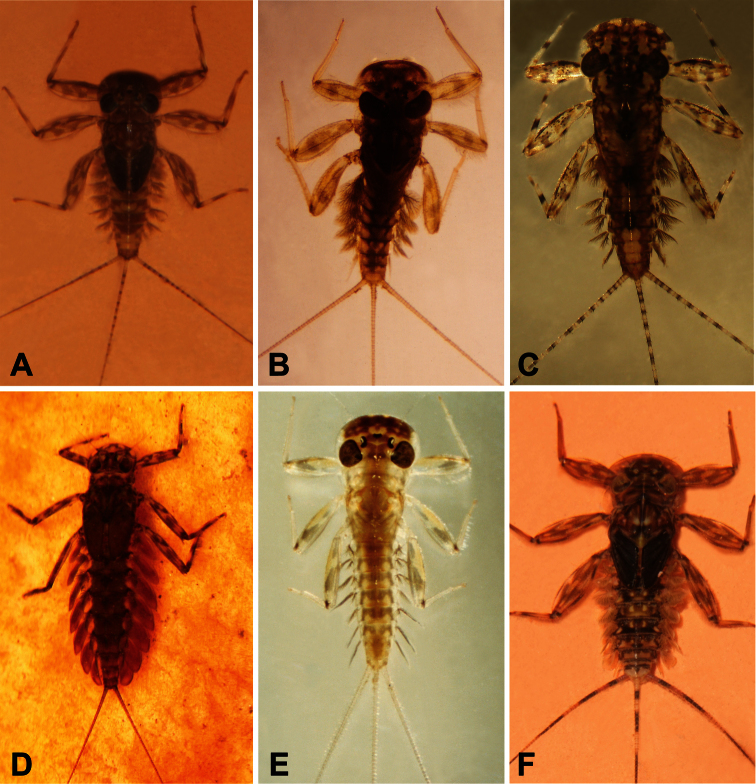
**A** Habitus of *Asionurus namnaoensis* Braasch & Boonsoong, 2010 **B** habitus of *Asionurus primus* Braasch & Soldán, 1986 **C**habitus of *Compsoneuria thienemanni*Ulmer, 1939 **D**habitus of *Epeorus khayengensis* Boonsoong & Braasch, 2010 **E** habitus of *Rhithrogena tonkinensis* Soldán & Braasch, 1986 **F** habitus of *Thalerosphyrus sinuosus* Navás, 1933.

*Thalerosphyrus sinuosus* larvae (Fig. 9F) are one of the most widespread mayfly species in Thailand. The larvae are found underneath stones in slow-flowing reaches of streams (water velocity approximately 3-7 cm/sec, water depth ranges between 7–17 cm). The larvae cling to submerged boulders and cobbles. Because they feed by grazing on diatoms, algae and detritus on stream rocks, they prefer rocky substrates in fairly clear to silty sediments.

#### Remarks.

Only one species of *Thalerosphyrus* (*Thalerosphyrus sinuosus*) was identified from Thailand and this species widely distributed in Thai streams. The larva and adults of this species were adequately described by Braasch and Soldán (1984b) and Navás (1933).

### Genus Compsoneuria

Eaton, 1881

http://species-id.net/wiki/Compsoneuria

Figs 2H 3E–L 7A–B 9C

#### Remarks.

The heptageniid mayfly genus *Compsoneuria* was reviewed by Webb et al. (2006). Eleven species were revised from Afro-tropical and Oriental regions. *Compsoneuria* larvae were found abundantly in lowland rivers on mainland Southeast Asia. There are three species of *Compsoneuria* mayflies reported from Thailand (Braasch 1990; Braasch 2006a; Braasch and Boonsoong 2010). *Compsoneuria* larvae occur among submerged vascular plants or roots.

### Compsoneuria thienemanni

Ulmer, 1939

http://species-id.net/wiki/Compsoneuria_thienemanni

Figs 3I–J 7A–B 9C

Compsoneuria thienemanni Ulmer, 1939: 672, Figs 440–448, 449–454. (orig.)
Compsoneuria thienemanni Braasch & Soldán, 1986b: 46. (comb.)
Thalerosphyrus thienemanni Wang & McCafferty, 2004: 17. (comb.)

#### Larva.

Ulmer 1939: 672, Figs 440–448, 449–454; Braasch and Soldán 1986b: 42–46, Figs 14.1–14.14.

#### Adult.

Ulmer 1939: 564, M, Figs 145–149, 152; F, 151; Fs, 150; Braasch and Soldán 1986b: 42–44, M, Figs 5-9.

#### Eggs.

Chorionic surface characterized by granular matrix, large KCTs randomly scattered on entire egg surface (Fig. 7A), small micropyles visible on equatorial plane (Fig. 7B).

#### Distribution.

Mae Hong Son province, Chiang Mai province, Trat province.

#### Diagnosis.

*Compsoneuria thienemanni* is recognised by having gills 2-6 without emarginations and gill 7 leaf-like and pointed apically; pale dots and marks on abdomen as shown in Fig. 3I.

#### Habitat and biology.

*Compsoneuria thienemanni* larvae (Fig. 9C) are found abundantly in the large rivers at lower altitudes where it is encountered clinging to floating submerged water plants. A few male specimens of *Compsoneuria thienemanni* were found in the mountainous region of Mae Hong Son province.

#### Remarks.

The larva and adults of this species were described by Ulmer (1939) and Braasch and Soldán (1986b). The larvae of this species are found abundantly in the low-gradient streams and large rivers in Thailand.

### Compsoneuria langensis

Braasch & Boonsoong, 2010

http://species-id.net/wiki/Compsoneuria_langensis

Figs 2H 3K–L

Compsoneuria langensis Braasch & Boonsoong, 2010: 9–11, Figs 31–45. (orig.)

#### Larva.

Braasch and Boonsoong 2010: 9–11, Figs 31–45.

**Adult.** Braasch and Boonsoong 2010: 7–9, M, Figs 19, 21–25, 27–30; F, Figs 20, 26.

#### Eggs.

Unknown.

#### Distribution.

Nam Lang River, Soppong (Mae Hong Son province).

#### Diagnosis.

Larva without emarginations on gills 2–6, gill 7 lanceolate and rounded apically, pale dots and marks on abdomen as on Fig. 3K.

#### Habitat and biology.

Both *Compsoneuria thienemanni* and *Compsoneuria langensis* larvaeare mainly found attached to floating water plants or thick pads of green algae.

#### Remarks.

Braasch and Boonsoong (2010) described this species from Thailand based on larval and imaginal specimens. The larva of this species is different from other known species by gills 2–6 without emarginations, gill 7 lanceolate and rounded apically.

### Compsoneuria (Siamoneuria) kovaci

Braasch, 2006

http://species-id.net/wiki/Compsoneuria_kovaci

Figs 3E–H

Compsoneuria (Siamoneuria) kovaci Braasch, 2006: 50–51, Figs 9–21. (orig.)

#### Remarks.

A brief comment on the appropriate placement of *Compsoneuria (Siamoneuria) kovaci* in Braasch and Boonsoong (2010) is required. Larval diagnosis of *Compsoneuria(Siamoneuria)* is in contradiction to that of *Compsoneuria*, namely in lacking the combination of long, sharply pointed supracoxal spurs, black spotting on the head capsule and femora, and narrow, apically pointed glossae. *Siamoneuria* cannot belong in the genus *Compsoneuria* but probably deserves its own status; it appears to be a morphospecies whose characters are not in accordance with other known genera. For a final definition, important missing details of mouthparts and eggs should be included. For now, we see it as a species as "INCERTAE SEDIS" (Braasch and Boonsoong 2010). So, further studies and more specimens are needed to clear taxonomic of this species.

**Larva.** Braasch 2006a: 50–51, Figs 9–21.

#### Adult.

Braasch 2006a: 49–51, M, Figs 1–8.

#### Eggs.

Unknown.

#### Distribution.

Nam Lang river, Soppong, Mae Hong Son province,

#### Diagnosis.

Larva is conspicuous due to emarginations on gills 2–6, gill 7 being narrowly lanceolate, the head without markings, and the body with paired paramedian spots on tergites 5–8, large median spot on 9, and three distantly arranged small spots at anterior margin of tergite 10.

#### Habitat and biology.

The habitat of the single larva found was submerged roots of a tree standing at the river bank.

### Genus Asionurus

Braasch & Soldán, 1986

http://species-id.net/wiki/Asionurus

Figs 4A–B 7C–D 9B

#### Remarks.

Three species of *Asionurus* mayflies have been reported from the Oriental region (Braasch 2011; Braasch and Soldán 1986a; Braasch and Soldán 1986b). Only *Asionurus primus* Braasch & Soldán, 1986 was collected and reported from northern Thailand. The identity of Vietnamese specimens of *Asionurus primus* (Braasch and Soldán 1986a) with those of northern Thailand (Sangpradub et al. 2002) is probable but needs confirmation by reared males from Vietnam.

### Asionurus primus

Braasch & Soldán, 1986

http://species-id.net/wiki/Asionurus_primus

Figs 4A–B 7C–D 9B

Asionurus primus Braasch & Soldán, 1986a: 155–158, Figs 1–13. (orig.)

#### Larva.

Braasch and Soldán 1986a: 155–158, Figs 1–13.

#### Adult.

Braasch and Boonsoong 2010: 5–7, M, Figs 14–17; F, Fig. 18.

#### Eggs.

General shape ovoid, both poles with large KCTs densely arranged (Fig. 7C), many microgranules densely scattered all over the surface of the chorion, macrogranules on equatorial plane (Fig. 7D), border well-defined by a thickened rim beset with tubercles, 5–6 micropyles on equatorial plane.

#### Distribution.

Chaiyaphum province, Mae Hong Son province, Chiang Mai province.

#### Diagnosis.

*Asionurus primus* differs from *Asionurus ulmeri* (Braasch and Soldán 1986a) by shorter and more pointed wings of hypopharynx and gill 7 with bulging anterior portion and acutely shaped apically. In *Asionurus ulmeri* the wings of hypopharynx are longer and have rounded ends whereas gill 7 is narrow and long and hardly extended anterioriorly.

#### Habitat and biology.

*Asionurus primus* larvae (Fig. 9B) are often the most abundant in small mountain streams of Thailand. Larvae live beneath rocks and debris. They were found together mostly with those of *Notacanthurus baei*. Larval habitat preference is similar to that of larvae of *Notacanthurus baei*.

#### Remarks.

Only one species of *Asionurus* (*Asionurus primus*) was identified from Thailand. The larva and adults of this species was described by Braasch and Soldán (1986a) and Braasch and Boonsoong (2010). The larva of this species found in small mountain streams of Thailand.

### Genus Rhithrogeniella

Ulmer, 1939

http://species-id.net/wiki/Rhithrogeniella

Figs 4C 8A–B 9E

#### Remarks.

Genus *Rhithrogeniella* is unique in having scaled caudal filaments with special arrangements of stout bristles and finer setae in the proximal portion; distal portion segments bear longer, stiffer setae and at articulations short bristles alternate with fine setae. These characters are similarly expressed in several *Nixe* spp. from Taiwan (Kang and Yang 1994). In view of the similarity in egg structure *Rhithrogeniella* is recently often identified as *Nixe* Flowers, 1980. However, Ulmer(1939) published *Rhithrogeniella ornata* from Sundaland which clearly has priority over *Nixe* Flowers, 1980.

### Rhithrogeniella ornata

Ulmer, 1939

http://species-id.net/wiki/Rhithrogeniella_ornata

Rhithrogeniella ornata Ulmer, 1939: 575–576, Figs 165–174. (orig.)

#### Adult.

Ulmer 1939: 575–576, M, Figs 165–166, 169; Ms, fig. 170, 173–174; F, Figs 171–172; Fs, Figs 167–168.

### Rhithrogeniella tonkinensis

Soldán & Braasch, 1986

http://species-id.net/wiki/Rhithrogeniella_tonkinensis

Figs 4C 8A–B 9E

Rhithrogeniella tonkinensis Soldán & Braasch, 1986: 203–210, Figs 1–18. (orig.)

#### Larva.

Soldán and Braasch 1986: 203–210, Figs 1–18.

#### Adult.

Soldán and Braasch 1986: 203 (F), 206, 210 (Ms, Figs 19–22); Braasch 1990: 11–12, M, Figs 17.1–17.4.

#### Eggs.

Egg ovoid, chorionic surface with mesh-like reticular ridges of a hexagonal structure, evenly covered with KCTs (Fig. 8A), micropyle slightly oval with inconspicuous marginal rim, 1–2 small micropyles visible on the equatorial area (Fig. 8B).

#### Distribution.

Chiang Mai province, Chaiyaphum province.

#### Diagnosis.

The larvae of *Rhithrogena tonkinensis* can be distinguished from those of other genera of Heptageniidae by the following combination of characters: the head is approximately as broad as the pronotum, without a median emargination and marginal bristles and by the presence of interfacing setae on the caudal filaments (Soldán and Braasch 1986). The latter are provided at rings with rather stout spines regularly alternating with fine setae. Segments of filaments are “scaled”. Larvae of the other Southeast Asian species *Rhithrogeniella ornata* Ulmer, 1939 are unknown.

#### Habitat and biology.

Larvae of *Rhithrogena tonkinensis* (Fig. 9E) occur in relative deep waters (30-40 cm) with slow currents and smaller stones or coarse sand on the bottom. Larvae are good swimmers, but prefer to remain attached to the stone surface rather than swimming (Soldán and Braasch 1986).

#### Remarks.

The larva and adults of *Rhithrogena tonkinensis* were adequately described by Soldán and Braasch (1986). Only *Rhithrogena tonkinensis* found in relative deep waters with slow currents of Thai streams.

### Genus Afronurus

Lestage, 1924

http://species-id.net/wiki/Afronurus

Figs 3A 4D–G 8C–D 9A

#### Remarks.

The synonymization of *Cinygmina* with *Afronurus* is recognized by Wang and McCafferty (2004), Kluge (2004), and Braasch and Freitag (2008). The genus *Afronurus* includes at least 43 species from the Oriental region (Braasch 1987, 1990, 2005, 2011; Braasch and Boonsoong 2010, Braasch and Soldán 1984a, 1987, Flowers and Pescador 1984, Braasch and Jacobus 2011, Kang and Yang 1994, Kimmins 1937, Nguyen and Bae 2003, Venkataraman and Sivaramakrishnan 1989, Zhou and Zheng 2003). It indicates the complicated situation in determining species of *Afronurus* in the Oriental; in Southeast Asia many species are known only by larvae, or described as adults with affiliation of larvae from the same locality; the rearing of species and genetic investigations will be the aim of a future research. *Afronurus* larvae are usually the dominant heptageniid benthic macro-invertebrates in Thai streams. In this study, we propose five described species of the genus *Cinygmina* Kimmins, 1937 = *Afronurus* Lestage, 1924.

### Afronurus cervina

Braasch & Soldán, 1984

http://species-id.net/wiki/Afronurus_cervina

Cinygmina cervina Braasch & Soldán, 1984: 196–197, 199, Figs 17–31. (orig.)
Afronurus cervina Braasch & Soldán, 1984 (comb.)

#### Larva.

Braasch and Soldán 1984a: 196–197, 199, Figs 17–31, Vietnam; no record in Thailand.

#### Adult.

Braasch and Soldán 1984a: Vietnam; 196–197, 199, M, Figs 14–16; Braasch 1990: 8, Fs & Ms, Thailand.

#### Eggs.

Unknown.

#### Distribution.

Ban Nam Tok (Chiang Rai province).

#### Diagnosis.

Braasch (1990) reported this species based on male, female and subimago male and female specimens, and the head of a presumed larva of *Afronurus cervina* without markings; gill 1 somewhat upturned, narrowly banana-shaped (Figs 17–18, Braasch and Soldán 1984).

#### Habitat and biology.

This species is found to be an inhabitant of fast flowing rivers in Vietnam.

#### Remarks.

The larva and adults of *Afronurus cervina* were described by Braasch and Soldán (1984a). Only adults of *Afronurus cervina* found in Thailand. But, the larva of this species is not found in Thai streams**.**

### Afronurus dama

Braasch & Soldán, 1987

http://species-id.net/wiki/Afronurus_dama

Cinygmina dama Braasch & Soldán, 1987: 125, Figs 7.1–7.4. (orig.)
Afronurus dama Braasch & Soldán, 1987 (comb.)

#### Larva.

Braasch and Soldán 1987: Vietnam; 125, Figs 7.1–7.4.

#### Adult.

Braasch and Soldán 1987: 123, 125, 126, M, Figs 8.1–8.3; Braasch 1990: 8 (reported 2 M and 1 F from Thailand).

#### Eggs.

Unknown.

#### Distribution.

Nam Tok Ban Du (Chiang Rai province).

#### Diagnosis.

Head with blurred spots at forward margin; gill 1 up-turned banana-shape, 3 smoothly triangular gills with obliquely attached projection.

#### Habitat and biology.

The larvae of *Afronurus dama* were found in streams of Tam Dao, Song Dan, Vinh Puh province, Vietnam.

#### Remarks.

Only adults of *Afronurus dama* were reported from Thailand (Braasch 1990)**.** The larva and adults of *Afronurus dama* were described by Braasch and Soldán (1987).

### Afronurus gilliesiana

Braasch, 1990

http://species-id.net/wiki/Afronurus_gilliesiana

Fig. 4F

Cinygmina gilliesiana Braasch, 1990: 8, 10, Figs 13.1–13.4, 14–16. (orig.)
Afronurus gilliesiana Braasch, 1990 (comb.)

#### Larva (F).

Braasch 1990: 8, 10, Figs 13.1–13.4, 14–16.

#### Adult.

Unknown.

#### Eggs.

Unknown.

#### Distribution.

Mae Sot district (Tak province).

#### Diagnosis.

The larva of *Afronurus gilliesiana* can be distinguished from congeners by the combination of the following characters: head with indistinct spots; gill 1 broadly lanceolate (Fig. 13.1, Braasch 1990); gill 3 widely rounded triangular (Fig. 13.2, Braasch 1990), gill 5 obliquely rounded triangular with small projection (Fig. 13.3, Braasch 1990), and broad asymmetrically oval gill 7 (Fig. 13.4, Braasch 1990).

#### Habitat and biology.

The larvae of *Afronurus gilliesiana* were found in headwater streams in northern Thailand.

#### Remarks.

Only larva of *Afronurus gilliesiana* was reported from northern part of Thailand (Braasch 1990)**.** The larva of *Afronurus gilliesiana* were described by Braasch (1990). The adults of *Afronurus gilliesiana* are unknown.

### Afronurus namnaoensis

Braasch & Boonsoong, 2010

http://species-id.net/wiki/Afronurus_namnaoensis

Figs 3A 4D 8C–D 9A

Afronurus namnaoensis Braasch & Boonsoong, 2010: 1–3, Figs 6–13. (orig.)

#### Larva.

Braasch and Boonsoong 2010: 1–3, Figs 6–13.

#### Adult.

Braasch and Boonsoong 2010: 2–3, M, Figs 1–4; F, fig. 5.

#### Eggs.

The egg chorion of *Afronurus namnaoensis* is decorated with granules and two kinds of KCTs: small KCTs concentrated at each pole and much larger oval KCTs located equatorially (Fig. 8C); micropyles have an ovoid to round sperm guide (Fig. 8D), visible in the equatorial area. The micropyle is interposed between adjacent equatorial KCTs.

#### Distribution.

Phromlaeng stream (Chaiyaphum province); Yakraue stream (Petchabun province); Nam Lang river, Pangmapa/Soppong (Mae Hong Son province); Chiang Mai province.

#### Diagnosis.

Male of *Afronurus namnaoensis* is separated from Vietnamese *Afronurus cervina* in lacking a median penial cone, by the less deeply notched lobal apex, and titillators curved laterally and in their more medial position. Vietnamese *Afronurus dama* presents a terminal apex of the penis slightly notched at the inner angles, whereas *Afronurus namnaoensis* is recognized by somewhat elevated corners on both sides of the apices. Larvae are recognizable by an unmarked forehead, pointed and weakly curved gill 1 and smoothly rounded triangular gill 5 with crosswise projection. Two Vietnamese species, *Afronurus meo* and *Afronurus mnong* (Nguyen & Bae, 2003) have gills lacking these projections (Figs 8–10, Figs 18–20, Nguyen and Bae 2003).

#### Habitat and biology.

Larvae of *Afronurus namnaoensis* (Fig. 9A) are probably the most abundant species on rocks and stones in Nam Lang River and elsewhere in current waters of northern and northeastern Thailand. These mayflies are an important food source for headwater stream fishes (*Cyclocheilichthys apogon*, *Devario regina*, *Opsarius pulchellus*, and *Rasbora rasbora*).

#### Remarks.

Braasch and Boonsoong (2010) described this species from Thailand based on nymphal and imaginal specimens, and deposited them in the ZMKU, Bangkok. The larvae of *Afronurus namnaoensis* are the most abundant species in current waters of Thai streams.

### Afronurus rainulfiana

Braasch, 1990

http://species-id.net/wiki/Afronurus_rainulfiana

Fig. 4G

Cinygmina rainulfiana Braasch, 1990: 8, 10, 11, Figs 9–12, Figs 18.1–18.3. (orig.)
Afronurus rainulfiana Braasch, 1990 (comb.)

#### Larva (M).

Braasch 1990: 8, 10, 11, Figs 9–12, Figs 18.1–18.3.

#### Adult.

Unknown.

#### Eggs.

Unknown.

#### Distribution.

Mae Sot district (Tak province).

#### Diagnosis.

The larvae of *Afronurus rainulfiana* can be distinguished from congeners by the combination of the following characters: head with a distinct pattern of light spots (Fig. 9), a broad banana-shaped gill 1 (Fig. 18.1, Braasch 1990), asymmetrically oval gill 6 with sloping finger-like projection (Fig. 18.2, Braasch 1990) and gill 7 narrowly lanceolate (Fig. 18.3, Braasch 1990).

#### Habitat and biology.

The larvae of *Afronurus rainulfiana* were found in headwater streams.

#### Remarks.

Only larva of *Afronurus rainulfiana* was reported from northern part of Thailand and was described by Braasch (1990). The adults of *Afronurus rainulfiana* are unknown.

### Afronurus rubromaculata

You, Wu, Gui & Hsu, 1981

http://species-id.net/wiki/Afronurus_rubromaculata

Fig. 4E

Cinygmina rubromaculata You, Wu, Gui & Hsu, 1981: 4, Figs 1–13. (orig.)
Afronurus rubromaculata You, Wu, Gui & Hsu, 1981 (comb.)

#### Larva.

Wu et al. 1986: 67, Figs 1–10; Zhou and Zheng 2003: 757, Figs 7–10.

#### Adult.

You et al. 1981: 4, M & F, Figs 1–13; Zhou and Zheng 2003: 758, Fig. 17.

#### Eggs.

Unknown.

#### Distribution.

Ban Nam Tok (Chiang Rai province); Nam Lang river, Soppong, Mae Hong Son province.

#### Diagnosis.

This species is unique in the genus because of its abdominal pigmentation: terga pale yellow medially and reddish laterally. The male genitalia have an obvious projection between the two lobes. The larvae of this species are larger and have more pale dots and marks on head and body than those of the other known species (Figs 7, 9, 10, Zhou and Zheng 2003), gill 5 or 6 are provided with a small, thin projection (Fig. 8).

#### Habitat and biology.

Larvae of *Afronurus rubromaculata* were the only representatives of *Afronurus* encountered in the large river Mekong in February 2002 along the Thai-Laos border in the utmost north of Thailand. It is regularly found as a resident together with the dominant *Afronurus namnaoensis* on stones and rocks in Nam Lang River, altitude 600 m (Braasch 2006). This species is also found in Vietnam.

#### Remarks.

The larva and adults of *Afronurus rubromaculata* were adequately described by You et al. (1981), Wu et al. (1986) and Zhou and Zheng (2003). Only larva of *Afronurus rubromaculata* was reported in Thailand (Braasch and Boonsoong 2010).

## A key to the eggs of known genera and species of Heptageniidae in Thailand

**Table:** 

1	KCTs absent (Fig. 5A)	*Epeorus khayengensis* Boonsoong & Braasch
−	KCTs present	2
2	Small KCTs densely concentrated at each pole, much larger KCTs equatorially (Fig. 8C)	*Afronurus namnaoensis* Braasch & Boonsoong
−	KCTs not as above	3
3	Chorion tuberculate or with peg-like structures	4
–	Chorion reticulate (Fig. 8A)	*Rhithrogena tonkinensis* Soldán & Braasch
4	Coils concentrated at one or both poles and evenly distributed about remainder of egg (Fig. 5C, 6C, 7C)	5
−	Coils never concentrated at poles; evenly distributed around entire egg (Fig. 6A, 7A)	7
5	Coils concentrated at one pole; chorion surface with peg-like structure (Fig. 5C)	*Rhithrogena siamensis* Braasch & Boonsoong
−	Coils concentrated at both poles; chorion surface tuberculate	6
6	Many microgranules densely scattered all over the surface of the chorion; micropyle border well defined by a thickened rim beset with tubercles (Fig. 7C)	*Asionurus primus* Braasch & Soldán
−	Rounded tubercles are scattered all over the surface of the chorion; micropyle border not strongly thickened (Fig. 6C)	*Thalerosphyrus sinuosus* Navás
7	Chorionic surface folded, with many densely scattered crenulated granules; small KCTs covering the entire egg surface (Fig. 6A)	*Notacanthurus baei* Braasch & Boonsoong
−	Chorionic surface with many sizes of granular matrix; large KCTs randomly scattered (Fig. 7A)	*Compsoneuria thienemanni* Ulmer

## Conclusions and recommendations

Heptageniidae is the most diverse and abundant mayfly family in Thailand. The total number of Thai Heptageniidae described to date amounts to 9 genera and 22 species. The results presented here show that the Thai heptageniid fauna is dominated by Oriental genera. In addition, three Southeast Asian endemic genera morphospecies (*Asionurus, Siamoneuria* and *Trichogenia*) are found in Thailand. The species of the genera *Afronurus* and *Thalerosphyrus* are the most abundantly and widely distributed species found in Thai streams. Early studies of Thai heptageniid mayflies provide an important base on which to continue the study of these insects. It is important, now, to investigate the fauna further, which will lead to a better biogeographical understanding, and, at the same time, to begin a study of the biology and ecology, which is still very limited for species recorded in Thailand. Data on the ecology of heptageniids in Asian streams is limited. Dudgeon (1996) gives some preliminary data on life histories of five Hong Kong species, and estimates of their secondary production. Boonsoong (2002) presents some preliminary data on life history and diet of *Afronurus* species from Nam Nao National Park. These larvae show a non-seasonal multivoltine. Based on gut analyses, larvae are non-selective generalists. They feed mainly on detritus and diatoms and could be categorized as scrapers. Larvae of heptageniid mayflies often occur in reaches of fast-flowing streams where mixed substrates are composed of cobble, pebble, and gravel. In general, the distribution of heptageniid larvae depends upon substrate type and water current.

In addition to basic taxonomic research, revision of unclear or poorly defined genera, and association of larval and adult stages by rearing, investigation priorities of the Thai Heptageniidae can be summarized briefly as follows: study of life cycle and ecology of individual species distribution; of heptageniid larvae with respect to different water conditions.

## Supplementary Material

XML Treatment for Epeorus

XML Treatment for Epeorus (Belovius) khayengensis

XML Treatment for Epeorus (Belovius) unicornutus

XML Treatment for Epeorus (Iron) martinus

XML Treatment for Epeorus (Iron) thailandensis

XML Treatment for Epeorus (Epeorus) aculeatus

XML Treatment for Epeorus (Epeorus) bifurcatus

XML Treatment for Epeorus (Epeorus) inthanonensis

XML Treatment for Rhithrogena

XML Treatment for Rhithrogena (Tumungula) siamensis

XML Treatment for Trichogenia

XML Treatment for Trichogenia maxillaris

XML Treatment for Notacanthurus

XML Treatment for Notacanthurus baei

XML Treatment for Thalerosphyrus

XML Treatment for Thalerosphyrus sinuosus

XML Treatment for Compsoneuria

XML Treatment for Compsoneuria thienemanni

XML Treatment for Compsoneuria langensis

XML Treatment for Compsoneuria (Siamoneuria) kovaci

XML Treatment for Asionurus

XML Treatment for Asionurus primus

XML Treatment for Rhithrogeniella

XML Treatment for Rhithrogeniella ornata

XML Treatment for Rhithrogeniella tonkinensis

XML Treatment for Afronurus

XML Treatment for Afronurus cervina

XML Treatment for Afronurus dama

XML Treatment for Afronurus gilliesiana

XML Treatment for Afronurus namnaoensis

XML Treatment for Afronurus rainulfiana

XML Treatment for Afronurus rubromaculata
